# Pattern of patients’ attendance to the dental clinic of federal college of dental technology and therapy, Enugu, Nigeria

**DOI:** 10.11604/pamj.2018.29.151.14563

**Published:** 2018-03-14

**Authors:** Nneka Kate Onyejaka, Bode Nojeem Lawal, Robinson Anayo Okechukwu, Magnus Osakpemwoya Osayande, Ijeoma Chinwe Alamba

**Affiliations:** 1 Department of Child Dental Health, University of Nigeria, Enugu, Nigeria; 2 Department of Clinical Services, Federal College of Dental Technology and Therapy, Enugu, Nigeria

**Keywords:** Pattern, dental, attendance, Enugu

## Abstract

**Introduction:**

Oral health is part of general health and should not be considered in isolation, as it contributes to the individual's health related quality of life. The study aimed at assessing the pattern of attendance to the dental clinic using dental records of patients.

**Methods:**

This was a retrospective study of 6008 retrieved case notes from the dental clinic of Federal College of Dental Technology and Therapy, Enugu, from June, 2016 to May, 2017. Data on age, sex and occupation were retrieved from the dental records of the institution. Association between age, sex, occupation and patient flow to dental clinic was determined.

**Results:**

More females (55.7%) than males (44.3%) attended the dental clinic within the study period. Also, those aged 16 to 30 years (39.5%) and school pupils/students (40.8%) attended dental clinic the most when compared to other attendees. Patient flow was greatest in the first quarter of the year (27.1%), followed by the third quarter (26.1%). However, patient flow was least in the last quarter (20.9%). There was significant association between age (P < 0.001), occupation (P = 0.03) and patient flow to dental clinic in yearly quarters.

**Conclusion:**

Being an adolescent or young adult and being a student were significantly associated with patient flow to the dental clinic in yearly quarters in the study area. Patient flow was greatest in the first quarter and least in the last quarter of the year. There is need to increase dental awareness, especially for preventive visit among older age group and non-students in the populace.

## Introduction

Oral health is defined as the standard of oral and related issues which enables an individual to eat, speak and socialize without active disease, discomfort or embarrassment and which contributes to the general wellbeing of the individual [1]. It is part of general health and should not be considered in isolation, as it contributes to the individual's health related quality of life [2]. Oral health awareness is low in Nigeria [3] hence, more effort needs to be applied to create better awareness and ultimately increase dental clinic attendance. One of the ways to access health behavior is dental clinic attendance, [4] which may be for curative or preventive purposes. Some known factors that affect attendance at dental clinics include socio-economic status, [5] attitude towards dental care, [6] family structure [7, 8] cost [9] and time [10]. Other factors include culture [11] and ethnicity [12]. In a prior study, few children and more females attended the dental clinic when compared to adults and males respectively [4]. A few studies on pattern of attendance to dental clinics [4, 13] based in tertiary institutions have been conducted in the area, but none on secondary or primary dental care centres. The services rendered by the different levels of dental care centres vary and this may influence the pattern of attendance to the centres. This study identified the pattern of attendance to the dental clinic of Federal College of Dental Technology and Therapy which is a secondary dental care institution. It also assessed the association between age, sex, occupation and patient flow to the dental clinic.

## Methods

**Study area**: The study area was the Federal College of Dental Technology and Therapy (FCDTT), Trans-Ekulu, in Enugu East Local Government Area (LGA) of Enugu State, Nigeria. It is situated in Enugu metropolis. Nigeria is made up of 36 states and six geopolitical zones. Enugu State is in the South Eastern geopolitical zone of the country. It is located in the 6.30' north and 7.30' east of the equator and has a population of 3.3 million [14] The inhabitants are mainly civil servants, traders and farmers. FCDTT has a dental clinic where patients are managed by dental surgeons. The college trains dental technologists and therapists who attend to patients referred to them by the dental surgeons. Students in the department of dental therapy are trained on the oral hygiene of patients referred to them by the dental surgeons, while students in the department of dental technology are involved in rehabilitative care of patients seen by the dental surgeons.

**Study design and study population**: This was a retrospective study in which data was extrapolated from the record book of the dental clinic of FCDTT. Dental attendance was defined as visit to the dentist to utilize preventive and/or curative services with procedures documented in the case notes. The independent variables were age, sex and occupation while the dependent variable was patient flow to dental clinic in yearly quarters.

**Data collection tool**: A case record form was developed to record the retrieved data of all patients who attended the dental clinic within the official working hours of the clinic for the one year study period. Data on age, sex and occupation of the study participants who attended the dental clinic was collected from the retrieved case notes.

**Study procedure**: After due consideration of ethical issues, data were extrapolated from the record book by the researchers. Data on age, sex and occupation of the study participants were recorded. All incomplete records were excluded from the study while those with complete records were included.

**Data handling**: Data were kept confidential by numbering the participants' case record form instead of using names. Information on patient flow for the year was grouped into four (first quarter: January to March; second quarter: April to June; third quarter: July to September; fourth quarter: October to December) for ease of analysis. Occupation was grouped as 'students', 'civil servants', 'private workers', 'unemployed', 'clergy'.

**Data analysis**: Statistical Package of Social Science (SPSS version 17) was used to analyze the data generated. Exploratory analysis was conducted to ensure data consistency. Descriptive analysis was conducted to determine the proportion of patients who attended the dental clinic according to age, sex and occupation. Chi square analysis was also conducted to determine the association between age, sex, occupation and patient flow to dental clinic. P values <0.05 were considered to be statistically significant.

## Results

**Figure 1:**
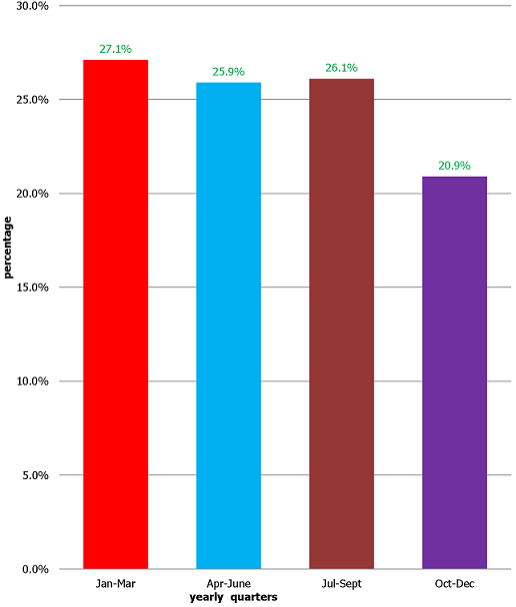
Pattern of patient flow to dental clinic of FCDTT in yearly quarters

A total of 6008 dental records with complete data were retrieved for the study. The age range was 1 year to 102 years. The mean age was 33.6 ± 18.2years. More female patients 3344 (55.7%), patients aged 16 to 30 years 2373(39.5%) and students 2459(40.8%) attended the dental clinic. (Table 1). There was significant association between age (P<0.001), occupation (P = 0.03) and patient flow to the dental clinic in yearly quarters. Patients aged 60 years and above 602(10.0%) and clergymen 81(1.3%) had poor attendance to the dental clinic. However, there was no statistically significant association between sex and patient flow to the dental clinic in yearly quarters (P = 0.83) Table 2. Patient flow to the dental clinic was highest in the first quarter (January to March) of the year (27.1%) and least (20.9%) in the fourth quarter as seen in Figure 1.

**Table 1:** General characteristics of patients (N = 6008)

Variables	FrequencyN (%)
**Age(years)**	
≤ 15	826 (13.7)
16 - 30	2,373 (39.5%)
31 - 45	1,367 (22.8)
46 - 60	840 (14.0)
≥ 60	602 (10.0)
Total	6,008 (100.0)
**Sex**	
male	2,664 (44.3)
female	3,344 (55.7)
Total	6,008 (100.0)
**Occupation**	
Students	2,450 (40.8)
Civil servants	1,237 (20. 6)
Private workers	663 (11.0)
Unemployed	1,577 (26.2)
Clergymen	81 (1.3)
**Total**	6,008 (100.0)

**Table 2:** Association between age, sex, occupation and patient flow to dental clinic in yearly quarters (N = 6008)

Variable	1^st^ quarterN (%)	2^nd^ quarterN (%)	3^rd^ quarter(N%)	4^th^ quarter(N%)	TotalN (%)	P value
**Age(years)**						
≤ 15	200 (12.3)	201 (12.9)	275 (17.5)	150 (11.9)	826 (13.7)	< 0.001
16 - 30	661 (40.6)	605 (38.9)	582 (37.1)	525 (41.8)	2,373 (39.5)	
31 - 45	407 (25.0)	363 (23.3)	327 (20.9)	270 (21.5)	1,367 (22.8)	
46 - 60	198 (12.1)	223 (14.3)	228 (14.6)	191 (15.2)	840 (14.0)	
≥ 60	164 (10.1)	163 (10.5)	155 (9.9)	120 (9.6)	602 (10.0)	
Total	1,630 (100.0)	1,555 (100.0)	1,567 (100.0)	1,256 (100.0)	6,008 (100.0)	
**Sex**						
Male	737 (45.2)	691 (44.4)	687 (43.8)	549 (43.9)	2,664 (44.3)	0.83
Female	893 (54.8)	864 (55.6)	880 (56.2)	707 (56.1)	3,344 (55.7)	
Total	1,630 (100.0)	1,555 (100.0)	1567(100.0)	1,256 (100.0)	6,008 (100.0)	
**Occupation**						0.03
Students	642 (39.4)	613 (39.4)	679 (43.3)	516 (41.1)	2,450 (40.8)	
Civil servants	331 (20.3)	321 (20. 6)	327 (20.9)	258 (20. 5)	1,237 (20. 6)	
Private workers	466 (28.6)	413 (26.6)	395 (25.2)	303 (24.1)	1,577 (26.2)	
Unemployed	164 (10.1)	187 (12.0)	151 (9.6)	11 (12.8)	663 (11.0)	
Clergymen	27 (1.7)	21 (1.4)	15 (1.0)	18 (1.4)	81 (1.3)	
**Total**	1,630 (100.0)	1,555 (100.0)	1,567 (100.0)	1,256 (100.0)	6,008 (100.0)	

## Discussion

The study showed significant association between being an adolescent or young adult, being a student and patient flow to the dental clinic in yearly quarters. Flow was greatest in the first quarter and least in the last quarter of the year. This study had the limitation of being a retrospective study which is characterized by incomplete records. However, the large sample size added to the validity of the findings of the study. Also, the study area being in the metropolis reduced the negative effect of far distance on accessing health facilities by patients, unlike a prior study in which the dental facility for the study was far from metropolis residential area.[4]. Hence, it was possible to see a true picture of the pattern of dental attendance in this study. Utilization of dental services is important for the wellbeing of an individual because underutilization can lead to poor oral health, with its attendant impact on the individual's quality of life [15]. Untreated dental diseases like dental caries might lead to dental pain and impact on the daily activities of children and adolescents in terms of play, sleep, eating and school activities [16]. In this study, adolescents and younger adults (16 to 30 years) utilized the dental services the most. This is in line with a previous study [17] and may be attributed to the fact that they are more conscious of their appearance and may also have more incidence of dental caries which is the commonest dental disease observed in younger age groups [17, 18]. Children's visit to the dental clinic was low as also reported in prior studies [4, 10]. Misinterpretation of dental problems in children by their care givers and non-availability of time for caregivers to take children to dental clinics are some of the reasons attributed to this [10, 19]. Poor attendance to dental clinic was also recorded by the elderly, despite the fact that they are the most rapidly growing segment of the population and oral health is essential to their general health and well-being [20]. Students, who were supposed to be in school, attended the dental clinic the most, probably because of the interplay between their age and dental caries; which is their commonest dental problem. Untreated caries is often associated with pain, infection, difficulty in chewing, premature tooth loss, speech difficulties, [21] school absenteeism and poor performance in school [22]. Inability to get away from work may be the reason for the low turnout of patients from other occupations. The operational time of most government dental clinics is same as regular office hours. The last quarter of the year witnessed the least flow of patients to the dental clinic. This could be as a result of the habitual movement of people to their villages to celebrate the Christmas and New Year festivals in the south eastern part of the country. The first quarter of the year witnessed a high turnout of patients, contrary to findings in a prior study in which the second quarter had the greatest turn out of patients [4]. This might be because many patients who returned to the city after the celebrations attended the dental clinic for their dental needs. This has implications on booking appointments with patients, especially towards the festive period because these appointments may not be kept. Future study on the reasons for presentation to the dental clinic in the study environment is recommended.

## Conclusion

There was significant association between being an adolescent or young adult, being a student and patient flow to the dental clinic in yearly quarters in the study area. There is need to increase awareness of dental health so that every age group and people engaged in various occupations can attend the dental clinic and access both preventive and curative care.

### What is known about this topic

- More females than males attend the dental clinic;
- Few younger children usually attend the dental clinic.

### What this study adds

- Adolescents and young adults attended the dental clinic the most while older patients attended the least. There is need to increase dental awareness among the elderly;
- The dental clinic was attended mostly by students, suggesting that school oral health programmes will be of benefit in the study environment;
- Patient flow was greatest in the first quarter of the year and least in the last quarter. This has implications for booking appointments. Patients may fail to keep appointments in the last quarter of the year while the dentists may get overworked in the first quarter of the year.

## Competing interests

The authors declare no conflict of interests.
